# The contribution of common mental disorders and alcohol-related morbidity to educational differences in early labour market exit among older workers: a register-based cohort study

**DOI:** 10.1093/eurpub/ckae212

**Published:** 2025-01-11

**Authors:** Emma Carlsson, Tomas Hemmingsson, Jonas Landberg, Bo Burström, Emelie Thern

**Affiliations:** Department of Public Health Sciences, Stockholm University, Stockholm, Sweden; Department of Global Public Health, Karolinska Institutet, Stockholm, Sweden; Department of Public Health Sciences, Stockholm University, Stockholm, Sweden; Institute of Environmental Medicine, Karolinska Institutet, Stockholm, Sweden; Department of Public Health Sciences, Stockholm University, Stockholm, Sweden; Department of Clinical Neuroscience, Karolinska Institutet, Stockholm, Sweden; Department of Global Public Health, Karolinska Institutet, Stockholm, Sweden; Institute of Environmental Medicine, Karolinska Institutet, Stockholm, Sweden

## Abstract

Previous studies have identified educational differences in early labour market exits, yet the mechanisms behind these disparities remain unclear. This study aims to examine to what extent common mental disorders (CMD) and alcohol-related morbidity can explain educational differences in early labour market exit. This cohort study included all men born 1951–53 who underwent conscription examination for military service in Sweden at age 18–20 (*n* = 136 466). The highest level of educational attainment and early labour market exit, using five different exit routes, was obtained from nationwide registers. Mediation analysis was used to examine the contribution of CMD and alcohol-related morbidity to the educational differences in early labour market exit. Factors measured in childhood, late adolescence, and early adulthood were included as confounders. Lower-educated men were at higher risk of leaving the labour market early. CMD contributed marginally to the educational differences in early exit due to disability pension, long-term sickness absence, and long-term unemployment, explaining up to 4%. Alcohol-related morbidity explained up to 12% of the educational differences in disability pension, long-term sickness absence, and long-term unemployment. Neither CMD nor alcohol-related morbidity were associated with early old-age retirement with and without income. Alcohol-related morbidity appears to be of importance when trying to understand educational differences in some but not all early labour market exit routes. Thus, reducing the negative effects of alcohol consumption could reduce educational inequalities in early exits from the labour market and prolong working life for all individuals regardless of socioeconomic position.

## Introduction

Socioeconomic inequalities in early exits from the labour market are well-known. Those with lower socioeconomic positions (SEP) are at higher risk of leaving the labour market before normative retirement age [1, 2], often due to involuntary reasons such as poor health [2, 3]. In Sweden, the labour force participation rate is in general high compared to other European countries. Still, almost 20% between the ages of 55–64 did not participate in the labour force in 2022 [4]. Previous research found poor general health and unfavourable working conditions to explain parts, but not all, of the educational differences found in early labour market exits [5–7]. However, we do not know how important mental health and alcohol-related morbidity are in explaining the educational differences in early exits. Yet, poor mental health [8, 9] as well as alcohol-related morbidity [10] has been found to be unequally distributed across SEP groups in the population, such that those in lower SEP are worse off. Additionally, these two factors are also associated with a higher risk of leaving the labour market early [11–14].

Poor mental health, including depressive and anxiety disorders, is one of the leading causes of burden of disease in the world [15]. Mental health is associated with a higher risk of leaving the labour market early due to disability pension [6, 7, 16–18], sickness absence [17, 18], and unemployment [6, 7, 16–18]. Studies show mixed results on the link between mental health and early retirement [6, 16, 17]. Furthermore, poor mental health has been found to contribute to explaining the educational differences in early labour market exit, but the magnitude of the contribution tends to differ in previous research [7, 17, 18]. Additionally, according to previous studies, poor mental health was found to be more prevalent among the lower educated [6, 7, 16–18], which could potentially explain educational differences in early exits.

Another leading cause of disease and death is alcohol [19]. Alcohol consumption and problematic drinking are linked to higher unemployment risk, especially among the less educated [20]. However, other studies did not find an association between alcohol consumption and early exit, such as disability pension, unemployment, or early retirement [5], and only a borderline association with sickness absence [21]. In these previous studies, those with lower educational levels had a higher risk of a higher alcohol intake [5, 20, 21]. However, research studying educational differences in early labour market exit exploring the role of alcohol-related morbidity is scarce.

Previous research found that factors established in early life, such as childhood SEP and cognitive ability in adolescence, are important to consider when examining educational differences in early labour market exit [17, 18, 22]. Considering that this early selection into education can also partly determine later-life health [23], these early-life factors need to be accounted for as possible confounders when assessing the contribution of risk factors to early labour market exits later in life. However, research examining educational differences in early exits and the role of mental health and alcohol while considering early life factors, prior mental illness, and alcohol consumption is rare [18].

Against this background, this study aimed to examine to what extent common mental disorders (CMD) and alcohol-related morbidity could explain educational differences in early labour market exit. To this end, we utilized a large cohort of men using nationwide registers and focused on five different exit routes. To enhance the robustness of our findings, the analyses were adjusted for potential confounding factors measured in childhood, late adolescence, and early adulthood.

## Methods

This cohort study, based on registry data, included all men born between 1951 and 1953 who underwent the conscription examination for military service in Sweden when they were 18–20 years old (*n* = 167 499). At that time, the conscription examination was mandatory for the entire Swedish male population at this age, and the data covers at least 90% of this population [24]. This study includes all men registered in Sweden at age 55. Men who died or received disability pension before age 55, and individuals lacking educational information, were excluded from the analysis (*n* = 31 033). Excluded men had lower educational attainment, lower childhood SEP, poorer health during late adolescence and early adulthood, and were more frequently diagnosed with CMD or alcohol-related morbidity during adulthood (Supplementary Table S1). The final analytical sample comprised 136 466 men (see Supplementary Fig. S1). Ethical approval was obtained by the Ethical Review Authority in Stockholm, reference number 2019-02161.

### Level of education (exposure)

The highest level of educational attainment was obtained when the men were 54 years old, using data from the Longitudinal Register of Education and Labour Market Statistics (LISA) [25]. Educational attainment was classified into five distinct groups: ≤9 years (primary school), 10–11 years (1–2 years of upper secondary), 12 years (3 years of upper secondary), 13–14 years (1–2 years of university), and ≥15 years (at least 3 years of university). Although educational attainment was measured at age 54, most men completed their education before 30. Therefore, we assume education precedes CMD and alcohol-related morbidity.

### Early labour market exit (outcome)

Early labour market exit was measured from 55 years of age (1 January 2006 at the earliest) up until the year the men turned 64 years old (up to 31 December 2017), using the LISA register. Five early exit routes were defined and analysed separately: disability pension, long-term sickness absence, long-term unemployment, early old-age retirement with income, and early old-age retirement without income. In Sweden, the Swedish Social Insurance Agency can grant disability pensions to individuals aged 30–65 years who have a medically verified disease or injury resulting in reduced work capacity [25]. For this study, disability pension was defined as receiving either full- or part-time disability pension benefits, in line with previous research [22, 26]. Long-term sickness absence was defined as receiving sickness benefits from the Swedish Social Insurance Agency for a minimum of 90 days per year over two consecutive years. Similarly, long-term unemployment was defined as being registered as full-time unemployed for a minimum of 180 days per year over two consecutive years, documented by the Swedish Public Employment Service. Two consecutive years were chosen to identify men more distance from the labour market as opposed to men in shorter periods of sickness absence and unemployment [22]. In Sweden, early old-age retirement can be granted from the age of 61 years. Early old-age retirement with income was defined as taking any old-age pension during a given year and earning an income exceeding one Price Base Amount (PBA) [27] (∼4500 euros per year) the following year [22, 26]. Conversely, early old-age retirement without income was defined as taking any old-age pension during a given year and earning an income below one PBA the subsequent year [22, 26]. For the analyses of early old-age retirement with and without income, men who either passed away or received disability pension between the ages 55–61 were excluded from the sample (*n* = 5896).

### CMD and alcohol-related morbidity (mediators)

Diagnoses for CMD and alcohol-related morbidity were obtained from when the men were 30 years old (1 January 1981 at the earliest) up until the year the men turned 54 years old (31 December 2007 at the longest) using inpatient data from the National Patient Register [28]. The Swedish versions of the International Classification of Diseases (ICD) version 8 (1974–86), 9 (1987–96), and 10 (1997 and onwards) were used. CMD included depressive disorders (ICD-8: 296, 298, 299; ICD-9: 296; ICD-10: F32–F34), anxiety disorders (ICD-8: 300, 790; ICD-9: 300; ICD-10: F40–F42), and stress-related disorders (ICD-9: 308, 209; ICD-10: F43). Alcohol-related morbidity included alcohol dependency (ICD-8: 291, 303, 979; ICD-9: 291, 303; ICD-10: F10), alcoholic liver disease (ICD-8: 571; ICD-9: 571; ICD-10: K70), and toxic effect of alcohol (ICD-8: 980; ICD-9: 980; ICD-10: T51). Both main and contributing diagnoses were used to define the mediators.

### Childhood, late adolescence, and early adulthood (confounders)

The inclusion of confounders was based on previous research [17, 18, 22]. Mental disorders and alcohol-related morbidity in early adulthood were included to distinguish early cases from those diagnosed in mid-adulthood, ensuring accurate temporality with educational attainment.

Parental educational attainment was used as a measure of childhood SEP. These data were extracted from the 1970 National Population and Housing Census when the men were between the ages of 17–19. The highest parental education was used and categorized like the exposure.

In late adolescence, the men underwent the conscription examination and several factors were assessed, described in detail elsewhere [24]. In this study, we included cognitive ability (low, medium, high), stress resilience (low, medium, high), body mass index (<25 or ≥25), mental disorders (ICD-8; 290-315), and musculoskeletal diagnoses (ICD-8; 710-738).

Early mental disorders and early alcohol-related morbidity were measured from the year 1974 (when the men were 21–23 years old) up until the men were 29 years old (up to 31 December 1982). This information was obtained from inpatient data in the National Patient Register. Early mental disorders included the ICD-8 codes 290-315 and early alcohol-related morbidity included the ICD-8 codes 291, 303, 571, 979, and 980.

Missing values on the confounders (due to lack of data in the registers and internal missing data) were coded as separate categories since analysing complete cases yielded similar results (Supplementary Tables S2 and S3).

### Statistical analysis

Differences in baseline characteristics between groups were estimated using Pearson’s chi-square tests. The association between CMD and alcohol-related diagnoses and each outcome was estimated using a generalized linear model with a log-binomial regression. For all outcomes, each model was run separately, therefore an individual could be observed in more than one outcome.

In order to estimate the mediating role of CMD and alcohol-related morbidity, we employed the potential outcomes framework [29]. The total effect of education on the outcome was decomposed into direct and indirect effects. The direct effect captures the effect of education on the outcome independently of the mediator, while the indirect effect captures the effect of education on the outcome through the mediator [30]. The *mediate* command in Stata 18 was used for the mediation analysis and separate models were fitted for each outcome and mediator. The models were specified as logistic models to estimate risk ratios with 95% confidence intervals (CIs) for each educational category, with ≥15 years as the reference group. For each outcome and mediator, a crude model and a model adjusted for all confounding factors are presented. The proportion of the total effect attributable to the indirect effect was quantified as proportion-mediated {calculated as [(NDE × (NIE-1))/(NDE × NIE-1)] × 100}. The total effect results from multiplying the indirect effect by the direct effect. All men, including those who died or emigrated during follow-up, were included in the analysis. The main reason for including these men was to avoid bias, as their deaths or emigrations might be related to the outcomes and not at random [31]. A sensitivity analysis was conducted after the exclusion of these men which yielded similar results as in the main analysis (Supplementary Tables S4 and S5). All analyses were accomplished using Stata Statistical Software (StataCorp LLC, College Station, TX, USA).

## Results

As seen in Table 1, men with lower levels of education had lower childhood SEP, worse health in late adolescence and in early adulthood, compared to higher-educated men. In adulthood, lower-educated men were diagnosed with CMD and alcohol-related morbidity more frequently, compared to higher-educated men.

**Table 1.** Baseline characteristics of the study population, stratified by years of education, using row percentage for the total sample and column percentage for the baseline characteristics

Years of education	≥15, *n* (%)	13–14, *n* (%)	12, *n* (%)	10–11, *n* (%)	≤9, *n* (%)	*P*-value
Total	23 872 (17.5)	19 837 (14.5)	19 987 (14.7)	42 078 (30.8)	30 692 (22.5)	
*Childhood and late adolescence*						
Parental education						<0.001
≤9	8505 (35.6)	10 025 (50.5)	11 147 (55.8)	27 692 (65.8)	23 043 (75.1)	
10–11	4595 (19.3)	4097 (20.7)	3955 (19.8)	7488 (17.8)	3566 (11.6)	
12	2994 (12.5)	2219 (11.2)	1791 (9.0)	2416 (5.7)	1010 (3.3)	
13–14	1677 (7.0)	931 (4.7)	625 (3.1)	730 (1.7)	282 (0.9)	
≥15	4287 (18.0)	1215 (6.1)	963 (4.8)	544 (1.3)	222 (0.7)	
Missing	1814 (7.6)	1350 (6.8)	1506 (7.5)	3208 (7.6)	2569 (8.4)	
Cognitive ability						<0.001
High	14 040 (58.8)	8743 (44.01)	6818 (34.1)	6271 (14.9)	2598 (8.5)	
Medium	7807 (32.7)	9165 (46.2)	10 255 (51.3)	24 234 (57.6)	15 560 (50.7)	
Low	512 (2.1)	929 (4.7)	1803 (9.0)	9137 (21.7)	10 547 (34.4)	
Missing	1513 (6.3)	1000 (5.0)	1111 (5.6)	2436 (5.8)	1987 (6.5)	
Stress resilience						<0.001
High	8228 (34.5)	6163 (31.1)	5229 (26.2)	7412 (17.6)	4445 (14.5)	
Medium	10 958 (45.9)	10 117 (51.0)	10 698 (53.5)	23 282 (55.3)	16 140 (52.6)	
Low	3088 (12.9)	2496 (12.6)	2882 (14.4)	8789 (20.9)	7998 (26.0)	
Missing	1598 (6.7)	1061 (5.3)	1177 (5.9)	2595 (6.2)	2109 (6.9)	
BMI ≥ 25	793 (3.3)	944 (4.8)	1002 (5.0)	2698 (6.4)	2426 (7.9)	<0.001
Missing	2218 (9.3)	1596 (8.1)	1660 (8.3)	3920 (9.3)	2875 (9.4)	
Mental disorders	2173 (9.1)	1724 (8.7)	1911 (9.6)	5764 (13.7)	5144 (16.8)	<0.001
Musculoskeletal diagnoses	3564 (14.9)	3095 (15.6)	3096 (15.5)	6909 (16.4)	5131 (16.7)	<0.001
*Early adulthood*						
Early mental disorders	130 (0.5)	126 (0.6)	147 (0.7)	472 (1.1)	315 (1.0)	<0.001
Early alcohol-related morbidity	23 (0.1)	36 (0.2)	44 (0.2)	254 (0.6)	183 (0.6)	<0.001
*Adulthood*						
Common mental disorders	364 (1.52)	318 (1.60)	343 (1.72)	932 (2.21)	633 (2.06)	<0.001
Alcohol-related morbidity	220 (0.92)	250 (1.26)	394 (1.97)	1170 (2.78)	953 (3.11)	<0.001
*Outcome*						
Disability pension	655 (2.74)	703 (3.54)	862 (4.31)	2564 (6.09)	2064 (6.72)	<0.001
Long-term sickness absence	1052 (4.41)	1149 (5.79)	1386 (6.93)	3881 (9.22)	2937 (9.57)	<0.001
Long-term unemployment	675 (2.83)	737 (3.72)	843 (4.22)	2137 (5.08)	1341 (4.37)	<0.001
Early old-age retirement with income	3403 (14.64)	3343 (17.39)	3160 (16.43)	7237 (18.12)	5335 (18.45)	<0.001
Early old-age retirement without income	4976 (21.41)	5432 (28.25)	5833 (30.33)	12 235 (30.63)	9224 (31.90)	<0.001

During follow-up, 6848 (5%) men exited the labour market through disability pension, 10 405 (8%) through long-term sickness absence, 5733 (4%) through long-term unemployment, 22 478 (17%) through early old-age retirement with income, and 37 700 (29%) through early old-age retirement without income. Early labour market exit, due to all of the five exit routes, was more frequently seen among the lower compared to the higher-educated men (Table 1).

Both CMD and alcohol-related morbidity were associated with an increased risk of leaving the labour market early through disability pension, long-term sickness absence, and long-term unemployment (see Table 2). However, neither CMD nor alcohol-related morbidity was found to be associated with an increased risk of early old-age retirement with and without income (and therefore, mediation analysis for these two outcomes was not conducted). On the contrary, alcohol-related morbidity was associated with a small decreased risk of early old-age retirement with income.

**Table 2.** Unadjusted risk ratios and 95% confidence intervals on the association between common mental disorders and alcohol-related morbidity and each outcome separately

	Disability pension	Long-term sickness absence	Long-term unemployment	Early old-age retirement with income	Early old-age retirement without income
Common mental disorders	4.00 (3.68–4.35)	2.81 (2.60–3.03)	1.76 (1.53–2.03)	0.93 (0.85–1.03)	1.01 (0.94–1.08)
Alcohol-related morbidity	3.77 (3.48–4.09)	2.51 (2.33–2.72)	2.70 (2.43–3.00)	0.87 (0.79–0.96)	1.06 (0.99–1.13)

Compared to the men with the highest level of education, men with lower levels of education had an increased risk of early exit through all three exit routes included in the mediation analyses (see Tables 3 and 4). Men with the lowest level of education had a 2.45-fold (95% CI 2.25–2.67) higher risk of exit through disability pension, a 2.17-fold (95% CI 2.03–2.36) higher risk of exit through long-term sickness absence, and a 1.55-fold (95% CI 1.41–1.69) higher risk of exit through long-term unemployment compared to the highest educated men, in the crude models.

**Table 3.** Decomposition of the total effect of education on early exit into direct effect and indirect effect using common mental disorders as mediator^a^

Years of education	**≥15** **RR (95% CI)**	**13–14** **RR (95% CI)**	%Δ	**12** **RR (95% CI)**	%Δ	**10–11** **RR (95% CI)**	%Δ	**≤9** **RR (95% CI)**	%Δ
Disability pension (6848 events, 5.02%)									
Model 1—crude									
Total effect	1.00	1.29 (1.16–1.43)		1.57 (1.42–1.74)		2.22 (2.04–2.42)		2.45 (2.25–2.67)	
Natural direct effect	1.00	1.29 (1.16–1.43)		1.56 (1.42–1.73)		2.18 (2.00–2.37)		2.42 (2.22–2.63)	
Natural indirect effect	1.00	1.00 (0.99–1.01)	1	1.01 (0.99–1.01)	1	1.02 (1.01–1.02)	2	1.01 (1.01–1.02)	2
Model 2—adjusted									
Total effect	1.00	1.24 (1.12–1.38)		1.44 (1.30–1.59)		1.80 (1.64–1.97)		1.84 (1.68–2.03)	
Natural direct effect	1.00	1.24 (1.12–1.37)		1.43 (1.29–1.58)		1.77 (1.62–1.94)		1.82 (1.66–2.00)	
Natural indirect effect	1.00	1.00 (0.99–1.01)	1	1.00 (0.99–1.01)	2	1.02 (1.01–1.02)	4	1.01 (1.01–1.02)	3
Long-term sickness absence (10 405 events, 7.62%)									
Model 1—crude									
Total effect	1.00	1.31 (1.21–1.43)		1.57 (1.46–1.70)		2.09 (1.96–2.24)		2.17 (2.03–2.36)	
Natural direct effect	1.00	1.31 (1.21–1.42)		1.57 (1.45–1.70)		2.07 (1.94–2.11)		2.15 (2.01–2.31)	
Natural indirect effect	1.00	1.00 (0.99–1.01)	1	1.00 (0.99–1.01)	1	1.01 (1.01–1.01)	2	1.01 (1.00–1.01)	2
Model 2—adjusted									
Total effect	1.00	1.25 (1.15–1.36)		1.44 (1.33–1.56)		1.75 (1.63–1.88)		1.72 (1.60–1.86)	
Natural direct effect	1.00	1.25 (1.15–1.36)		1.44 (1.33–1.55)		1.73 (1.61–1.86)		1.71 (1.58–1.85)	
Natural indirect effect	1.00	1.00 (0.99–1.00)	1	1.00 (0.99–1.01)	1	1.01 (1.01–1.01)	2	1.01 (1.00–1.01)	2
Long-term unemployment (5733 events, 4.20%)									
Model 1—crude									
Total effect	1.00	1.31 (1.19–1.46)		1.49 (1.35–1.65)		1.80 (1.65–1.96)		1.55 (1.41–1.69)	
Natural direct effect	1.00	1.31 (1.19–1.46)		1.49 (1.35–1.65)		1.79 (1.64–1.95)		1.54 (1.41–1.69)	
Natural indirect effect	1.00	1.00 (0.99–1.00)	0	1.00 (0.99–1.00)	0	1.00 (1.00–1.01)	1	1.00 (1.00–1.01)	1
Model 2—adjusted									
Total effect	1.00	1.32 (1.19–1.47)		1.46 (1.32–1.62)		1.63 (1.49–1.79)		1.34 (1.21–1.48)	
Natural direct effect	1.00	1.32 (1.19–1.47)		1.46 (1.32–1.62)		1.63 (1.48–1.79)		1.33 (1.20–1.48)	
Natural indirect effect	1.00	1.00 (0.99–1.00)	0	1.00 (0.99–1.00)	0	1.00 (1.00–1.01)	1	1.00 (1.00–1.00)	1

a Crude (Model 1) and adjusted (Model 2) risk ratios (RRs) with 95% confidence intervals (CIs). Adjusted models are adjusted for all variables measured in childhood, late adolescence, and early adulthood. The proportion of the total effect that is due to the indirect effect is presented as proportion mediated (%Δ).

**Table 4.** Decomposition of the total effect of education on early exit into direct effect and indirect effect using alcohol-related morbidity as mediator^a^

Years of education	**≥15** **RR (95% CI)**	**13–14** **RR (95% CI)**	%Δ	**12** **RR (95% CI)**	%Δ	**10–11** **RR (95% CI)**	%Δ	**≤9** **RR (95% CI)**	%Δ
Disability pension (6848 events, 5.02%)									
Model 1—crude									
Total effect	1.00	1.29 (1.16–1.43)		1.57 (1.42–1.74)		2.22 (2.04–2.42)		2.45 (2.25–2.67)	
Natural direct effect	1.00	1.28 (1.15–1.42)		1.53 (1.39–1.69)		2.13 (1.96–2.32)		2.33 (2.14–2.55)	
Natural indirect effect	1.00	1.01 (1.00–1.01)	4	1.03 (1.02–1.03)	7	1.04 (1.04–1.05)	8	1.05 (1.04–1.06)	8
Model 2—adjusted									
Total effect	1.00	1.24 (1.12–1.38)		1.44 (1.30–1.59)		1.80 (1.64–1.97)		1.84 (1.67–2.02)	
Natural direct effect	1.00	1.23 (1.11–1.37)		1.40 (1.27–1.55)		1.73 (1.58–1.90)		1.76 (1.60–1.93)	
Natural indirect effect	1.00	1.01 (1.00–1.01)	4	1.02 (1.02–1.03)	7	1.04 (1.03–1.05)	8	1.05 (1.04–1.05)	10
Long-term sickness absence (10 405 events, 7.62%)									
Model 1—crude									
Total effect	1.00	1.31 (1.21–1.43)		1.57 (1.46–1.70)		2.09 (1.96–2.24)		2.17 (2.03–2.36)	
Natural direct effect	1.00	1.31 (1.21–1.42)		1.55 (1.44–1.68)		2.05 (1.91–2.19)		2.11 (1.97–2.26)	
Natural indirect effect	1.00	1.00 (1.00–1.01)	2	1.01 (1.01–1.02)	4	1.02 (1.02–1.03)	4	1.03 (1.02–1.03)	5
Model 2—adjusted									
Total effect	1.00	1.25 (1.15–1.36)		1.44 (1.33–1.56)		1.75 (1.63–1.88)		1.72 (1.60–1.86)	
Natural direct effect	1.00	1.25 (1.15–1.35)		1.42 (1.31–1.53)		1.71 (1.59–1.84)		1.68 (1.55–1.81)	
Natural indirect effect	1.00	1.00 (1.00–1.01)	2	1.01 (1.01–1.02)	4	1.02 (1.02–1.03)	5	1.03 (1.02–1.03)	6
Long-term unemployment (5733 events, 4.20%)									
Model 1—crude									
Total effect	1.00	1.31 (1.19–1.46)		1.49 (1.35–1.65)		1.80 (1.65–1.96)		1.55 (1.41–1.69)	
Natural direct effect	1.00	1.31 (1.18–1.45)		1.47 (1.33–1.62)		1.75 (1.60–1.90)		1.49 (1.36–1.64)	
Natural indirect effect	1.00	1.01 (1.00–1.01)	2	1.02 (1.01–1.02)	5	1.03 (1.02–1.03)	6	1.03 (1.03–1.04)	9
Model 2—adjusted									
Total effect	1.00	1.33 (1.19–1.47)		1.46 (1.32–1.62)		1.64 (1.49–1.80)		1.34 (1.21–1.49)	
Natural direct effect	1.00	1.32 (1.19–1.46)		1.44 (1.30–1.60)		1.60 (1.47–1.76)		1.30 (1.18–1.44)	
Natural indirect effect	1.00	1.00 (1.00–1.01)	2	1.01 (1.01–1.02)	5	1.03 (1.02–1.03)	6	1.03 (1.02–1.04)	12

a Crude (Model 1) and adjusted (Model 2) risk ratios (RRs) with 95% confidence intervals (CIs). Adjusted models are adjusted for all variables measured in childhood, late adolescence, and early adulthood. The proportion of the total effect that is due to the indirect effect is presented as proportion mediated (%Δ).

### Common mental disorders

The decomposition of the total effect into direct and indirect effect with CMD as a mediator is shown in Table 3. CMD mediated 0%–4% of the association between level of education and disability pension, long-term sickness absence, and long-term unemployment, depending on the level of education.

### Alcohol-related morbidity

Alcohol-related morbidity mediated 4%–10% of the association between level of education and disability pension, 2%–6% of the association between level of education and long-term sickness absence, and 2%–12% of the association between level of education and long-term unemployment, depending on the educational level (see Table 4).

## Discussion

The results of this study indicate that educational differences in early labour market exit due to disability pension, long-term sickness absence, and long-term unemployment were partially attributed to alcohol-related morbidity. CMD played a marginal role in these differences. Furthermore, these two health-related factors were not found to be associated with early old-age retirement with and without income.

This study found that although CMD was associated with an increased risk for some early exit routes, it only contributed slightly to explaining the educational differences in disability pension, long-term sickness absence, and long-term unemployment. This is in line with previous research on disability pension [7, 17, 18] and long-term sickness absence [17, 18]. Regarding long-term unemployment, previous research found different results, where some are in line with our study [17, 18], while another study found poor mental health to explain a considerable part of the educational differences in unemployment [7]. Different findings may result from measurement differences, as the previous study included anyone reporting unemployment within a year, without specifying long-term unemployment, and mental health was self-reported as opposed to diagnose-based [7]. The different results may also be due to different welfare state expressions in different countries. In line with previous research, we did not find CMD to be a risk factor for early old-age retirement [6, 16, 17].

Our study found that alcohol-related morbidity explained up to 12% of the educational differences found in disability pension, long-term sickness absence, and long-term unemployment. This finding is a contribution to the research field, given that no previous research to our knowledge has previously looked into the explanatory role of alcohol-related morbidity. Furthermore, this study found alcohol-related morbidity to be a risk factor for disability pension, long-term sickness absence, and long-term unemployment. This is in line with one previous study that found high alcohol consumption to be a risk for unemployment [20]. Other previous studies found alcohol consumption to not be associated with disability pension and unemployment [5], as well as sickness absence [21]. Unlike previous studies that rely on self-reported alcohol consumption, our study uses alcohol-related diagnoses from inpatient care. These diagnoses represent long-term consequences of alcohol consumption and provide a different perspective on alcohol-related problems. Another explanation for these different results may be that the exit definitions are different, for example, unemployment and sickness absence that we defined as long-term but others used more short-term measurements [5, 21]. These short-term measurements include unemployment from day 1 [5] and 10 days of sickness absence [21] which may not represent exits from the labour market but could rather be shorter spells of absence. The current study, on the other hand, defined unemployment and sickness absence as long-term, stretching over 2 years, to more definitely capture those very marginalized in the labour market. In line with previous research, we did not find an association between alcohol-related morbidity and early old-age retirement with and without income [5].

Comparing CMD and alcohol-related morbidity, alcohol-related morbidity explained more of the educational differences in early exit from the labour market. Previous studies that included both a health measure and alcohol when examining educational differences in early exit did not include any earlier health or alcohol measurements [5, 21], making a comparison to previous studies difficult. One explanation for the difference found in this study could be that we found CMD to both be less prevalent among the men in this study as well as showing a smaller educational gradient compared to alcohol-related morbidity. Another explanation could be attributed to health-related selection of the men in the study. Since we only included men who were alive and who had not received disability pension before follow-up, some men might already have left the labour market before our follow-up started, due to poor health. This may be explained by the healthy-worker effect which suggests that workers are healthier compared to the general population since individuals have to be at least somewhat healthy to be employed [32]. We found that those excluded from the study population were more often diagnosed with both CMD and alcohol-related morbidity during adulthood as well as having mental disorders and alcohol-related morbidity during early adulthood (see Supplementary Table S1). However, among the excluded men, it was more common to receive a mental disorder diagnosis compared to an alcohol-related diagnosis in early adulthood as well as a mental diagnosis in late adolescence, compared to the included men. Thus, one possible explanation for why alcohol-related morbidity was found to be more important as an explanatory factor in this study, compared to CMD, could be that it takes a long time to develop an alcohol-related diagnosis. Alcohol dependency [33] as well as the toxic effect of alcohol [34] may emerge before age 30 but the total burden of alcohol-related morbidity increases with age up until age 60 [19] or 70 [34]. CMD, on the other hand, often emerges during late adolescence or in the early 20s [33]. Consequently, more men could have already left the labour market before the follow-up started due to CMD compared to alcohol-related morbidity.

### Strengths and limitations

A major strength of this study was the possibility of following a large cohort of men throughout their lives using nationwide registers, reducing the risk of attrition and self-report bias. Another major strength was the long follow-up time up until 65 years of age and the inclusion of five different early exit routes, which expands previous research. This is a strength since the underlying mechanisms explaining the educational differences in these different early exit routes seem to differ and this better reflects the early exit routes available today. Furthermore, we included factors measured in childhood and late adolescence which is important since these determine educational attainment through health selection [23]. We were also able to include mental disorders and alcohol-related morbidity measured in early adulthood. This is a strength since it differentiates the diagnoses given in early adulthood from the ones given from 30 years of age and onwards, making it possible to measure the effects from later adulthood specifically. This seems to be especially important when considering health problems related to alcohol consumption, where diagnoses given tend to vary depending on the age of the individual, with more severe alcohol problems emerging later in life [19, 34].

An obvious limitation is that this study only includes men. The conscription cohort that includes almost all men born in Sweden at the time was used to be able to include the factors measured in late adolescence, which are important for indirect selection into education. However, educational differences in early labour market exit do not seem to differ between men and women [1] even though women tend to receive disability pension and sickness benefits more often compared to men in Sweden [35]. On the other hand, it is also a strength that we examine men separately since previous studies investigated men and women collectively. Another limitation is the use of inpatient data which means that we could only include the most severe cases. This may lead to an underestimation of the explanatory role of CMD and alcohol-related morbidity. Additionally, we were not able to measure alcohol consumption in this study which is a limitation since alcohol consumption tends to differ depending on socioeconomic positions and could, in itself, be a risk factor for early labour market exit [20]. We could not disentangle CMD and alcohol-related morbidity from other potential mediators like working conditions, consequently working conditions were not included in the analysis.

Furthermore, mediation analysis requires several no-confounding assumptions [36]. Despite including factors from childhood to early adulthood, residual confounding cannot be ruled out. We analysed the mediators separately as our focal interest was the separate effect of each mediator, which is less robust than combined analysis due to unmeasured confounding that is also influenced by the exposure [37]. The handling of missing data may introduce bias, but the complete case results were consistent with the main analysis.

## Conclusion

This study demonstrates that alcohol-related morbidity contributes to explaining educational differences in early labour market exit due to disability pension, long-term sickness absence, and long-term unemployment. However, the contribution of CMD to educational differences in early exit was marginal and none of the two factors could explain educational differences in early old-age retirement. Improving health and especially reducing long-lasting consequences from alcohol consumption may be one way to reduce socioeconomic inequalities in early labour market exits and prolong working life for everyone.

## Supplementary Material

ckae212_Supplementary_Data

## Data Availability

The data underlying this article were provided by Statistics Sweden under license for this study. Data will be shared on reasonable request to the corresponding author and with permission of Statistics Sweden. Key points Previous studies have found socioeconomic inequalities in early exits from the labour market, where those in lower socioeconomic positions are at higher risk. However, the mechanisms shaping these inequalities are not fully known. Using nationwide register-based data from Sweden, we found alcohol-related morbidity to mediate the relationship between educational differences in early exits from the labour market through disability pension, long-term sickness absence, and long-term unemployment. Common mental disorders (CMD) contributed marginally to explain the educational differences in early labour market exit. Early labour market exit through early old-age retirement with and without income was not found to be associated with CMD or alcohol-related morbidity. Alcohol-related morbidity is an important factor for explaining the educational differences in disability pension, long-term sickness absence, and long-term unemployment. Reducing long-term consequences from alcohol consumption could decrease socioeconomic inequalities in early labour market exit and prolong working life.
