# Supplementary material for: The contribution of common mental disorders and alcohol-related morbidity to educational differences in early labour market exit among older workers: a register-based cohort study
Source: Eur J Public Health. 2025 Jan 11;35(1):65–71. doi: 10.1093/eurpub/ckae212 (PMC11832143; doi:10.1093/eurpub/ckae212)
Supplement: ckae212_Supplementary_Data [file ckae212_supplementary_data.docx]

**
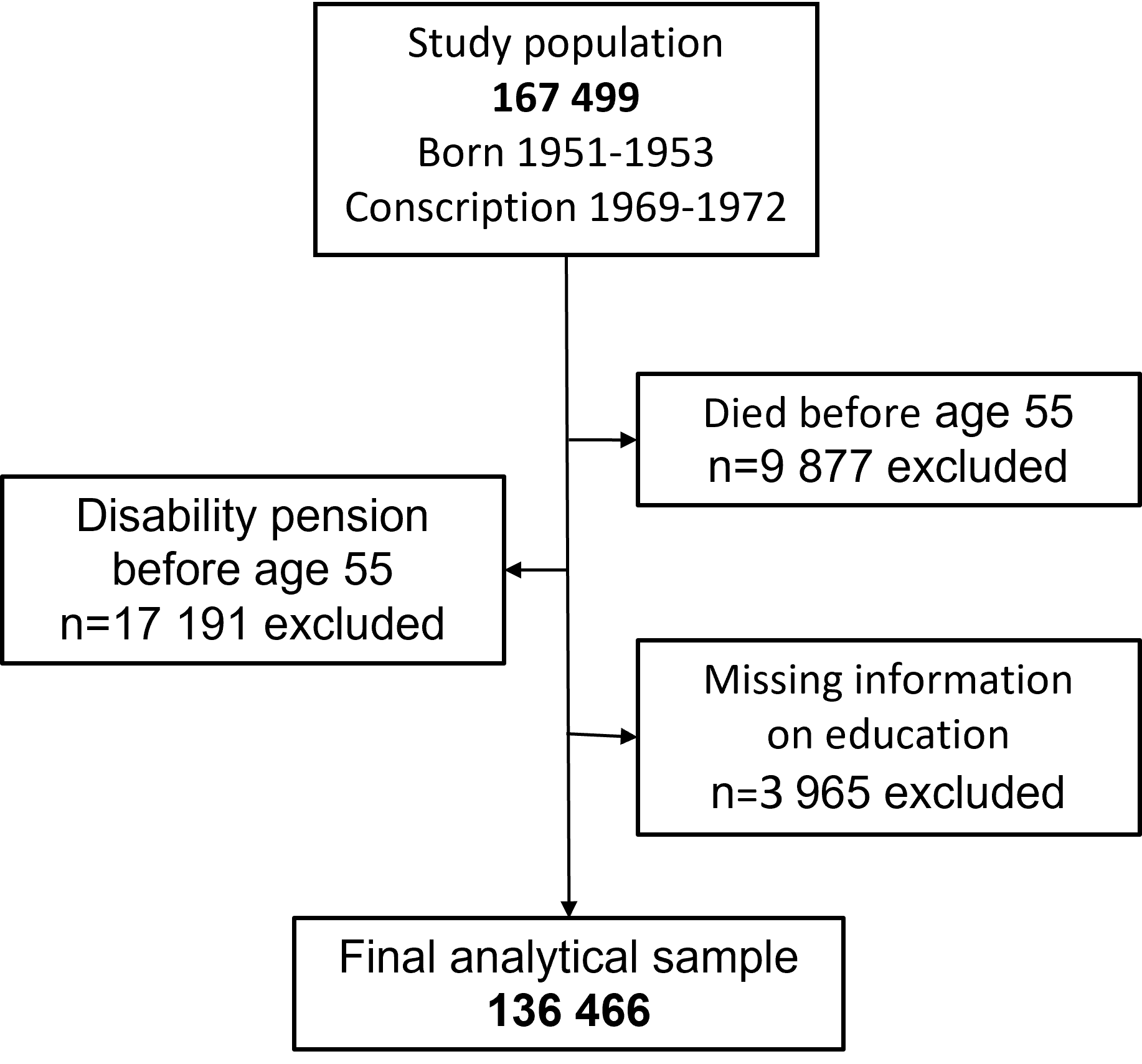
**

**Supplementary Figure 1.** Flow chart describing the selection process of the participants.

**Supplementary Table 1.** Baseline characteristics of the individuals included and excluded in the study population.

|  | **Included n (%)** | **Excluded n (%)** | **p-value** |
| --- | --- | --- | --- |
| **Total** | 136 466 (81.5) | 31 033 (18.5) |  |
| *Childhood and late adolescence* | |  |  |
| Parental education |  |  |  |
| ≤ 9 | 80 412 (58.9) | 17 545 (56.5) | <0.001 |
| 10-11 | 23 701 (17.4) | 4 634 (14.9) |  |
| 12 | 10 430 (7.6) | 1 903 (6.1) |  |
| 13-14 | 4 245 (3.1) | 716 (2.3) |  |
| ≥ 15 | 7 231 (5.3) | 1 246 (4.0) |  |
| Missing | 10 447 (7.7) | 4 989 (16.1) |  |
| Cognitive ability |  |  |  |
| High | 22 928 (16.8) | 7 486 (24.1) | <0.001 |
| Medium | 67 021 (49.1) | 12 134 (39.1) |  |
| Low | 38 470 (28.2) | 4 949 (16.0) |  |
| Missing | 8 047 (5.9) | 6 464 (20.8) |  |
| Stress resilience |  |  |  |
| High | 25 254 (18.5) | 8 785 (28.3) | <0.001 |
| Medium | 71 195 (52.2) | 11 543 (37.2) |  |
| Low | 31 477 (23.1) | 3 988 (12.9) |  |
| Missing | 8 540 (6.2) | 6 717 (21.6) |  |
| BMI ≥ 25 | 7 863 (5.8) | 2 061 (6.6) | <0.001 |
| Missing | 12 269 (9.0) | 7 432 (24.0) |  |
| Mental disorders | 16 716 (12.3) | 8 356 (26.9) | <0.001 |
| Musculoskeletal diagnoses | 21 795 (16.0) | 4 989 (16.1) | 0.6 |
| *Early adulthood* |  |  |  |
| Early mental disorders | 1 190 (0.9) | 2 073 (6.7) | <0.001 |
| Early alcohol-related morbidity | 540 (0.4) | 947 (3.1) | <0.001 |
| *Adulthood* |  |  |  |
| Common mental disorders | 2 590 (1.9) | 3 372 (10.9) | <0.001 |
| Alcohol-related morbidity | 2 987 (2.2) | 3 331 (10.7) | <0.001 |
| *Years of own education* |  |  |  |
| ≥ 15 | 23 872 (17.5) | 1 011 (3.2) | <0.001 |
| 13-14 | 19 837 (14.5) | 1 264 (4.1) |  |
| 12 | 19 987 (14.7) | 1 858 (6.0) |  |
| 10-11 | 42 078 (30.8) | 6 608 (21.3) |  |
| ≤ 9 | 30 692 (22.5) | 6 340 (20.4) |  |
| Missing | 0 | 13 952 (45.0) |  |

**Supplementary Table 2.** Complete case analysis excluding 18 236 individuals with missing information on factors measured in childhood and late adolescence. Mediation analysis with decomposition of the total effect of education on early exit into direct effect and indirect effect using common mental disorders as mediator. Crude (model 1) and adjusted (model 2) risk ratios with 95% confidence intervals. Adjusted models are adjusted for all variables measured in childhood, late adolescence and early adulthood. The proportion of the total effect that is due to the indirect effect is presented as proportion mediated (%Δ).

| **Years of education** | **≥ 15**  **RR (95% CI)** | **13-14**  **RR (95% CI)** | **%∆** | **12**  **RR (95% CI)** | **%∆** | **10-11**  **RR (95% CI)** | **%∆** | **≤ 9**  **RR (95% CI)** | **%∆** |
| --- | --- | --- | --- | --- | --- | --- | --- | --- | --- |
| **Disability pension (5 545 events, 4.69%)** | | | | | | | | | |
| Model 1 – crude |  |  |  |  |  |  |  |  |  |
| Total effect | 1.00 | 1.32 (1.18-1.49) |  | 1.59 (1.42-1.78) |  | 2.29 (2.08-2.52) |  | 2.54 (2.31-2.80) |  |
| Natural direct effect | 1.00 | 1.32 (1.18-1.48) |  | 1.58 (1.41-1.77) |  | 2.25 (2.05-2.47) |  | 2.50 (2.27-2.75) |  |
| Natural indirect effect | 1.00 | 1.00 (0.99-1.01) | 1 | 1.01 (0.99-1.02) | 2 | 1.02 (1.01-1.02) | 3 | 1.02 (1.01-1.02) | 3 |
| Model 2 – adjusted |  |  |  |  |  |  |  |  |  |
| Total effect | 1.00 | 1.24 (1.11-1.40) |  | 1.42 (1.27-1.59) |  | 1.80 (1.62-1.99) |  | 1.84 (1.65-2.05) |  |
| Natural direct effect | 1.00 | 1.24 (1.10-1.39) |  | 1.41 (1.26-1.58) |  | 1.77 (1.59-1.96) |  | 1.82 (1.63-2.02) |  |
| Natural indirect effect | 1.00 | 1.00 (0.99-1.01) | 1 | 1.00 (0.99-1.01) | 2 | 1.02 (1.01-1.02) | 3 | 1.01 (1.01-1.02) | 3 |
| **Long-term sickness absence (8 666 events, 7.33%)** | | | | | | | | | |
| Model 1 – crude |  |  |  |  |  |  |  |  |  |
| Total effect | 1.00 | 1.37 (1.26-1.50) |  | 1.63 (1.50-1.78) |  | 2.18 (2.02-2.35) |  | 2.33 (2.16-2.51) |  |
| Natural direct effect | 1.00 | 1.37 (1.26-1.50) |  | 1.63 (1.49-1.77) |  | 2.16 (2.00-2.32) |  | 2.30 (2.14-2.49) |  |
| Natural indirect effect | 1.00 | 1.00 (0.99-1.01) | 0 | 1.00 (0.99-1.01) | 1 | 1.01 (1.01-1.01) | 2 | 1.01 (1.01-1.01) | 2 |
| Model 2 – adjusted |  |  |  |  |  |  |  |  |  |
| Total effect | 1.00 | 1.29 (1.18-1.42) |  | 1.48 (1.35-1.61) |  | 1.80 (1.66-1.95) |  | 1.82 (1.67-1.98) |  |
| Natural direct effect | 1.00 | 1.29 (1.18-1.42) |  | 1.47 (1.35-1.61) |  | 1.78 (1.64-1.93) |  | 1.80 (1.65-1.96) |  |
| Natural indirect effect | 1.00 | 1.00 (0.99-1.01) | 0 | 1.00 (0.99-1.01) | 1 | 1.01 (1.01-1.01) | 2 | 1.01 (1.00-1.01) | 2 |
| **Long-term unemployment (4 663 events, 3.94%)** | | | | | | | | | |
| Model 1 – crude |  |  |  |  |  |  |  |  |  |
| Total effect | 1.00 | 1.32 (1.18-1.48) |  | 1.51 (1.35-1.68) |  | 1.82 (1.65-2.00) |  | 1.57 (1.42-1.74) |  |
| Natural direct effect | 1.00 | 1.32 (1.18-1.48) |  | 1.50 (1.35-1.68) |  | 1.81 (1.65-1.99) |  | 1.57 (1.42-1.73) |  |
| Natural indirect effect | 1.00 | 1.00 (0.99-1.00) | 0 | 1.00 (0.99-1.00) | 0 | 1.00 (1.00-1.01) | 1 | 1.00 (1.00-1.01) | 1 |
| Model 2 – adjusted |  |  |  |  |  |  |  |  |  |
| Total effect | 1.00 | 1.31 (1.17-1.47) |  | 1.46 (1.30-1.63) |  | 1.63 (1.47-1.81) |  | 1.35 (1.20-1.51) |  |
| Natural direct effect | 1.00 | 1.31 (1.17-1.47) |  | 1.45 (1.30-1.63) |  | 1.63 (1.46-1.81) |  | 1.34 (1.19-1.51) |  |
| Natural indirect effect | 1.00 | 1.00 (0.99-1.00) | 0 | 1.00 (0.99-1.00) | 0 | 1.00 (1.00-1.01) | 1 | 1.00 (1.00-1.00) | 1 |

**Supplementary Table 3.** Complete case analysis excluding 18 236 individuals with missing information on factors measured in childhood and late adolescence. Mediation analysis with decomposition of the total effect of education on early exit into direct effect and indirect effect using alcohol-related morbidity as mediator. Crude (model 1) and adjusted (model 2) risk ratios with 95% confidence intervals. Adjusted models are adjusted for all variables measured in childhood, late adolescence and early adulthood. The proportion of the total effect that is due to the indirect effect is presented as proportion mediated (%Δ).

| **Years of education** | **≥ 15**  **RR (95% CI)** | **13-14**  **RR (95% CI)** | **%∆** | **12**  **RR (95% CI)** | **%∆** | **10-11**  **RR (95% CI)** | **%∆** | **≤ 9**  **RR (95% CI)** | **%∆** |
| --- | --- | --- | --- | --- | --- | --- | --- | --- | --- |
| **Disability pension (5 545 events, 4.69%)** | | | | | | | | | |
| Model 1 – crude |  |  |  |  |  |  |  |  |  |
| Total effect | 1.00 | 1.32 (1.18-1.49) |  | 1.59 (1.42-1.78) |  | 2.29 (2.08-2.52) |  | 2.54 (2.31-2.80) |  |
| Natural direct effect | 1.00 | 1.31 (1.17-1.47) |  | 1.55 (1.38-1.73) |  | 2.18 (1.99-2.40) |  | 2.41 (2.19-2.66) |  |
| Natural indirect effect | 1.00 | 1.01 (1.00-1.02) | 4 | 1.03 (1.02-1.03) | 7 | 1.05 (1.04-1.06) | 8 | 1.05 (1.04-1.06) | 8 |
| Model 2 – adjusted |  |  |  |  |  |  |  |  |  |
| Total effect | 1.00 | 1.24 (1.11-1.40) |  | 1.42 (1.27-1.59) |  | 1.79 (1.62-1.99) |  | 1.84 (1.65-2.05) |  |
| Natural direct effect | 1.00 | 1.23 (1.10-1.39) |  | 1.39 (1.24-1.55) |  | 1.72 (1.55-1.91) |  | 1.75 (1.57-1.96) |  |
| Natural indirect effect | 1.00 | 1.01 (1.00-1.01) | 4 | 1.02 (1.02-1.03) | 8 | 1.04 (1.04-1.05) | 9 | 1.05 (1.04-1.06) | 10 |
| **Long-term sickness absence (8 666 events, 7.33%)** | | | | | | | | | |
| Model 1 – crude |  |  |  |  |  |  |  |  |  |
| Total effect | 1.00 | 1.37 (1.26-1.50) |  | 1.63 (1.50-1.78) |  | 2.18 (2.02-2.35) |  | 2.33 (2.16-2.51) |  |
| Natural direct effect | 1.00 | 1.37 (1.25-1.50) |  | 1.61 (1.48-1.75) |  | 2.12 (1.97-2.29) |  | 2.26 (2.09-2.44) |  |
| Natural indirect effect | 1.00 | 1.01 (1.00-1.01) | 2 | 1.01 (1.01-1.02) | 4 | 1.03 (1.02-1.03) | 5 | 1.03 (1.02-1.03) | 5 |
| Model 2 – adjusted |  |  |  |  |  |  |  |  |  |
| Total effect | 1.00 | 1.30 (1.18-1.42) |  | 1.48 (1.35-1.61) |  | 1.79 (1.65-1.95) |  | 1.81 (1.66-1.98) |  |
| Natural direct effect | 1.00 | 1.29 (1.18-1.41) |  | 1.46 (1.33-1.59) |  | 1.75 (1.61-1.90) |  | 1.76 (1.62-1.92) |  |
| Natural indirect effect | 1.00 | 1.00 (1.00-1.01) | 2 | 1.01 (1.01-1.02) | 4 | 1.02 (1.02-1.03) | 5 | 1.03 (1.02-1.03) | 6 |
| **Long-term unemployment (4 663 events, 3.94%)** | | | | | | | | | |
| Model 1 – crude |  |  |  |  |  |  |  |  |  |
| Total effect | 1.00 | 1.32 (1.18-1.48) |  | 1.51 (1.35-1.68) |  | 1.82 (1.65-2.00) |  | 1.57 (1.42-1.74) |  |
| Natural direct effect | 1.00 | 1.31 (1.17-1.47) |  | 1.48 (1.32-1.65) |  | 1.76 (1.60-1.93) |  | 1.51 (1.37-1.68) |  |
| Natural indirect effect | 1.00 | 1.01 (1.00-1.01) | 3 | 1.02 (1.01-1.02) | 5 | 1.03 (1.03-1.04) | 7 | 1.04 (1.03-1.05) | 10 |
| Model 2 – adjusted |  |  |  |  |  |  |  |  |  |
| Total effect | 1.00 | 1.32 (1.17-1.48) |  | 1.46 (1.30-1.64) |  | 1.64 (1.47-1.82) |  | 1.35 (1.20-1.52) |  |
| Natural direct effect | 1.00 | 1.31 (1.16-1.47) |  | 1.44 (1.28-1.61) |  | 1.59 (1.43-1.77) |  | 1.31 (1.16-1.47) |  |
| Natural indirect effect | 1.00 | 1.01 (1.00-1.01) | 2 | 1.02 (1.01-1.02) | 5 | 1.03 (1.02-1.04) | 7 | 1.03 (1.03-1.04) | 13 |

**Supplementary Table 4.** Mediation analysis excluding men who died or emigrated during follow-up (excluding 8 392 men, 6% of the total sample). Decomposition of the total effect of education on early exit into direct effect and indirect effect using common mental disorders as mediator. Crude (model 1) and adjusted (model 2) risk ratios with 95% confidence intervals. Adjusted models are adjusted for all variables measured in childhood, late adolescence and early adulthood. The proportion of the total effect that is due to the indirect effect is presented as proportion mediated (%Δ) (marked with bold if p-value <0.05).

| **Years of education** | **≥ 15**  **RR (95% CI)** | **13-14**  **RR (95% CI)** | **%∆** | **12**  **RR (95% CI)** | **%∆** | **10-11**  **RR (95% CI)** | **%∆** | **≤ 9**  **RR (95% CI)** | **%∆** |
| --- | --- | --- | --- | --- | --- | --- | --- | --- | --- |
| **Disability pension (5 993 events, 4.68%)** | | | | | | | | | |
| Model 1 – crude |  |  |  |  |  |  |  |  |  |
| Total effect | 1.00 | 1.28 (1.15-1.43) |  | 1.54 (1.38-1.71) |  | 2.18 (1.99-2.38) |  | 2.45 (2.24-2.69) |  |
| Natural direct effect | 1.00 | 1.28 (1.15-1.43) |  | 1.53 (1.38-1.70) |  | 2.14 (1.96-2.35) |  | 2.42 (2.21-2.65) |  |
| Natural indirect effect | 1.00 | 1.00 (0.99-1.01) | 0 | 1.01 (0.99-1.01) | 1 | 1.02 (1.01-1.02) | 3 | 1.01 (1.01-1.02) | 2 |
| Model 2 – adjusted |  |  |  |  |  |  |  |  |  |
| Total effect | 1.00 | 1.23 (1.10-1.38) |  | 1.40 (1.26-1.56) |  | 1.77 (1.61-1.95) |  | 1.84 (1.67-2.04) |  |
| Natural direct effect | 1.00 | 1.23 (1.10-1.37) |  | 1.40 (1.26-1.56) |  | 1.74 (1.58-1.92) |  | 1.82 (1.65-2.02) |  |
| Natural indirect effect | 1.00 | 1.00 (0.99-1.01) | 0 | 1.00 (0.99-1.01) | 1 | 1.01 (1.01-1.02) | 3 | 1.01 (1.01-1.02) | 2 |
| **Long-term sickness absence (9 273 events, 7.24%)** | | | | | | | | | |
| Model 1 – crude |  |  |  |  |  |  |  |  |  |
| Total effect | 1.00 | 1.31 (1.21-1.43) |  | 1.55 (1.42-1.68) |  | 2.11 (1.97-2.26) |  | 2.20 (2.04-2.36) |  |
| Natural direct effect | 1.00 | 1.31 (1.21-1.43) |  | 1.54 (1.42-1.68) |  | 2.09 (1.95-2.24) |  | 2.18 (2.03-2.34) |  |
| Natural indirect effect | 1.00 | 1.00 (0.99-1.01) | 0 | 1.00 (0.99-1.01) | 1 | 1.01 (1.01-1.01) | 2 | 1.01 (1.00-1.01) | 1 |
| Model 2 – adjusted |  |  |  |  |  |  |  |  |  |
| Total effect | 1.00 | 1.25 (1.15-1.36) |  | 1.41 (1.30-1.54) |  | 1.76 (1.63-1.90) |  | 1.73 (1.60-1.88) |  |
| Natural direct effect | 1.00 | 1.25 (1.15-1.36) |  | 1.41 (1.30-1.54) |  | 1.74 (1.61-1.88) |  | 1.72 (1.59-1.87) |  |
| Natural indirect effect | 1.00 | 1.00 (0.99-1.00) | 0 | 1.00 (0.99-1.01) | 1 | 1.01 (1.01-1.01) | 2 | 1.01 (1.00-1.01) | 2 |
| **Long-term unemployment (5 352 events, 4.18%)** | | | | | | | | | |
| Model 1 – crude |  |  |  |  |  |  |  |  |  |
| Total effect | 1.00 | 1.30 (1.17-1.44) |  | 1.49 (1.34-1.64) |  | 1.76 (1.62-1.92) |  | 1.51 (1.37-1.68) |  |
| Natural direct effect | 1.00 | 1.30 (1.17-1.44) |  | 1.48 (1.34-1.64) |  | 1.76 (1.61-1.92) |  | 1.50 (1.40-1.65) |  |
| Natural indirect effect | 1.00 | 1.00 (0.99-1.00) | 0 | 1.00 (0.99-1.00) | 0 | 1.00 (1.00-1.01) | 1 | 1.00 (1.00-1.00) | 1 |
| Model 2 – adjusted |  |  |  |  |  |  |  |  |  |
| Total effect | 1.00 | 1.31 (1.17-1.45) |  | 1.45 (1.31-1.61) |  | 1.61 (1.46-1.78) |  | 1.31 (1.18-1.45) |  |
| Natural direct effect | 1.00 | 1.31 (1.17-1.45) |  | 1.45 (1.31-1.61) |  | 1.60 (1.45-1.76) |  | 1.30 (1.17-1.45) |  |
| Natural indirect effect | 1.00 | 1.00 (0.99-1.00) | 0 | 1.00 (0.99-1.00) | 0 | 1.00 (1.00-1.00) | 1 | 1.00 (1.00-1.00) | 1 |

**Supplementary Table 5.** Mediation analysis excluding men who died or emigrated during follow-up (excluding 8 392 men, 6% of the total sample). Decomposition of the total effect of education on early exit into direct effect and indirect effect using alcohol-related morbidity as mediator. Crude (model 1) and adjusted (model 2) risk ratios with 95% confidence intervals. Adjusted models are adjusted for all variables measured in childhood, late adolescence and early adulthood. The proportion of the total effect that is due to the indirect effect is presented as proportion mediated (%Δ) (marked with bold if p-value <0.05).

| **Years of education** | **≥ 15**  **RR (95% CI)** | **13-14**  **RR (95% CI)** | **%∆** | **12**  **RR (95% CI)** | **%∆** | **10-11**  **RR (95% CI)** | **%∆** | **≤ 9**  **RR (95% CI)** | **%∆** |
| --- | --- | --- | --- | --- | --- | --- | --- | --- | --- |
| **Disability pension (5 993 events, 4.68%)** | | | | | | | | | |
| Model 1 – crude |  |  |  |  |  |  |  |  |  |
| Total effect | 1.00 | 1.28 (1.15-1.43) |  | 1.54 (1.38-1.71) |  | 2.18 (1.99-2.38) |  | 2.45 (2.24-2.69) |  |
| Natural direct effect | 1.00 | 1.27 (1.14-1.42) |  | 1.50 (1.35-1.67) |  | 2.11 (1.93-2.31) |  | 2.36 (2.15-2.58) |  |
| Natural indirect effect | 1.00 | 1.01 (1.00-1.01) | 3 | 1.02 (1.01-1.03) | 6 | 1.03 (1.03-1.04) | 6 | 1.04 (1.03-1.05) | 7 |
| Model 2 – adjusted |  |  |  |  |  |  |  |  |  |
| Total effect | 1.00 | 1.23 (1.10-1.38) |  | 1.40 (1.26-1.56) |  | 1.77 (1.60-1.94) |  | 1.83 (1.66-2.03) |  |
| Natural direct effect | 1.00 | 1.22 (1.10-1.37) |  | 1.38 (1.24-1.53) |  | 1.71 (1.56-1.89) |  | 1.77 (1.60-1.96) |  |
| Natural indirect effect | 1.00 | 1.01 (1.00-1.01) | 3 | 1.02 (1.02-1.02) | 6 | 1.03 (1.02-1.04) | 7 | 1.04 (1.03-1.04) | 8 |
| **Long-term sickness absence (9 273 events, 7.24%)** | | | | | | | | | |
| Model 1 – crude |  |  |  |  |  |  |  |  |  |
| Total effect | 1.00 | 1.31 (1.21-1.43) |  | 1.55 (1.42-1.68) |  | 2.11 (1.97-2.26) |  | 2.20 (2.04-2.36) |  |
| Natural direct effect | 1.00 | 1.31 (1.20-1.43) |  | 1.53 (1.41-1.66) |  | 2.07 (1.93-2.22) |  | 2.14 (1.99-2.30) |  |
| Natural indirect effect | 1.00 | 1.00 (1.00-1.01) | 2 | 1.01 (1.01-1.02) | 4 | 1.02 (1.02-1.02) | 4 | 1.03 (1.02-1.03) | 4 |
| Model 2 – adjusted |  |  |  |  |  |  |  |  |  |
| Total effect | 1.00 | 1.25 (1.15-1.36) |  | 1.41 (1.30-1.54) |  | 1.76 (1.63-1.90) |  | 1.73 (1.59-1.87) |  |
| Natural direct effect | 1.00 | 1.25 (1.14-1.36) |  | 1.40 (1.28-1.52) |  | 1.72 (1.60-1.86) |  | 1.69 (1.56-1.83) |  |
| Natural indirect effect | 1.00 | 1.00 (1.00-1.01) | 2 | 1.01 (1.01-1.02) | 4 | 1.02 (1.02-1.02) | 4 | 1.02 (1.02-1.03) | 5 |
| **Long-term unemployment (5 352 events, 4.18%)** | | | | | | | | | |
| Model 1 – crude |  |  |  |  |  |  |  |  |  |
| Total effect | 1.00 | 1.30 (1.17-1.44) |  | 1.49 (1.34-1.64) |  | 1.76 (1.62-1.92) |  | 1.51 (1.37-1.66) |  |
| Natural direct effect | 1.00 | 1.29 (1.16-1.43) |  | 1.46 (1.32-1.62) |  | 1.72 (1.58-1.88) |  | 1.47 (1.33-1.61) |  |
| Natural indirect effect | 1.00 | 1.00 (1.00-1.01) | 2 | 1.01 (1.01-1.02) | 4 | 1.02 (1.02-1.03) | 5 | 1.03 (1.02-1.04) | 9 |
| Model 2 – adjusted |  |  |  |  |  |  |  |  |  |
| Total effect | 1.00 | 1.31 (1.17-1.45) |  | 1.46 (1.31-1.62) |  | 1.61 (1.46-1.78) |  | 1.31 (1.18-1.46) |  |
| Natural direct effect | 1.00 | 1.30 (1.17-1.45) |  | 1.44 (1.29-1.60) |  | 1.58 (1.43-1.74) |  | 1.28 (1.15-1.42) |  |
| Natural indirect effect | 1.00 | 1.00 (1.00-1.01) | 2 | 1.01 (1.01-1.02) | 4 | 1.02 (1.02-1.03) | 6 | 1.03 (1.02-1.03) | 11 |
